# Supplementary material for: Efficacy and safety of Yunkang oral liquid combined with conventional therapy for threatened miscarriage of first-trimester pregnancy a protocol for systematic review and meta-analysis
Source: PLoS One. 2022 Feb 8;17(2):e0263581. doi: 10.1371/journal.pone.0263581 (PMC8824317; doi:10.1371/journal.pone.0263581)
Supplement: S1 File — (DOCX) [file pone.0263581.s002.docx]

Appendix 1. Search terms for ICTRP and ClinicalTrials.gov

Yunkang oral liquid AND miscarriage

Yunkang oral liquid AND abortion

CNKI

SU=孕康口服液*(先兆流产+流产)*(随机+对照+试验+分组)-(动物+大鼠+小鼠)

WANGFANG

主题:孕康口服液*(先兆流产+流产)*(随机+对照+试验+分组)-(动物+大鼠+小鼠)

VPN

主题:孕康口服液*(先兆流产+流产)

CBM

主题:孕康口服液*(先兆流产+流产)

PUBMED

#1 threatened miscarriage[mh] OR miscarriage[mh] OR threatened abortion[mh] OR abortion[mh] OR fetal loss[mh] OR pregnancy loss[mh]

#2 Yunkang oral liquid[mh]

#3 randomized controlled trial [pt] OR controlled clinical trial [pt] OR randomized [tiab] OR placebo [tiab] OR drug therapy [sh]OR randomly [tiab] OR trial [tiab] OR groups [tiab]

#4 human[mh] NOT animal[mh]

#5 #1 AND #2 AND #3 AND #4

EMBASE

#1 ('threatened miscarriage'/de OR 'threatened abortion'/de OR 'fetal loss'/de OR 'pregnancy loss'/de OR 'abortion'/exp OR 'abortion'/de OR 'pregnancy termination'/de OR (pregnant* AND terminat*) OR (abort* AND pregnant*) OR (abort* AND pregnanc*) OR (terminat* AND pregnanc*))

#2 ('Yunkang oral liquid'/de)

#3 ('crossover procedure':de OR 'double-blind procedure':de OR 'randomized controlled trial':de OR  'single-blind procedure':de OR (random* OR  factorial* OR crossover* OR cross NEXT/1 over* OR placebo* OR doubl* NEAR/1 blind* OR singl* NEAR/1 blind* OR assign* OR allocat* OR volunteer*):de,ab,ti)

#4 #1 AND #2 AND #3

Cochrane Library

#1 exp abortion, threatened/

#2 (abortion* or miscarriage*).mp.

#3 miscarriage. af

#4 threatened.af

5 3 and 4

6 1 or 2 or 5

7 exp Yunkang oral liquid

8 Yunkang oral liquid. af

9 7 or 8

10 5 and 9

Web of Science

#1 TS= (“threatened miscarriage”) OR (“threatened abortion”) OR (“fetal loss”) OR (“pregnancy loss”) OR (miscarriage) OR (abortion)

#2 TS=(“Yunkang oral liquid”)

#3 #1 AND #2
