# Supplementary material for: Efficacy and safety of Yunkang oral liquid combined with conventional therapy for threatened miscarriage of first-trimester pregnancy a protocol for systematic review and meta-analysis
Source: PLoS One. 2022 Feb 8;17(2):e0263581. doi: 10.1371/journal.pone.0263581 (PMC8824317; doi:10.1371/journal.pone.0263581)
Supplement: S2 File — (DOCX) [file pone.0263581.s003.docx]

# The product information of Yunkang oral liquid

About the composition of the Yunkang oral liquid and how these were reported in the original studies, produced by Echobe Group Zhejiang Qiqi Pharmaceutical Co., Ltd. Therefore, we listed them as one row.


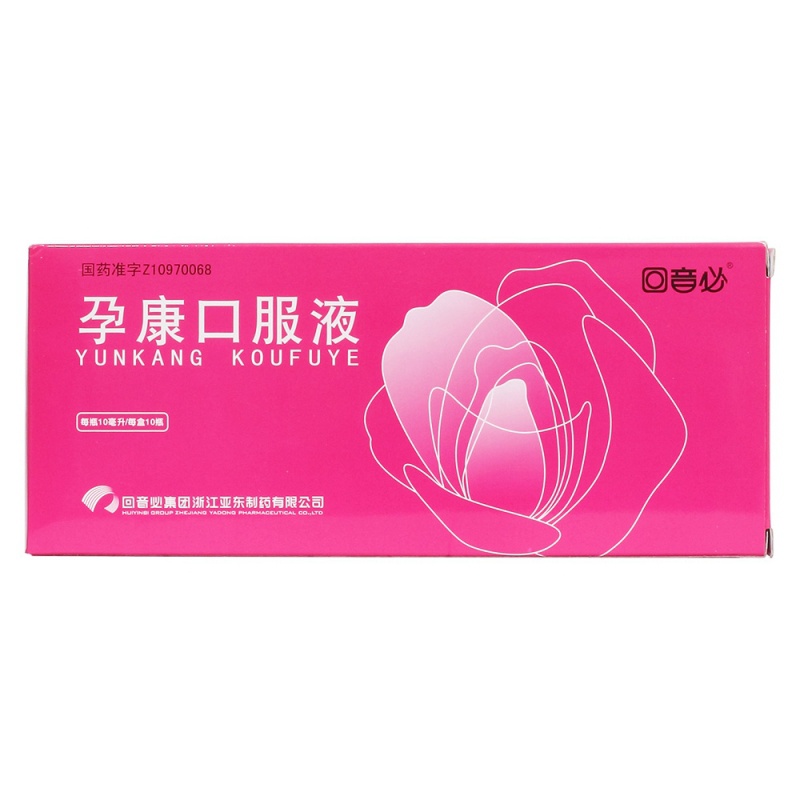


**Figure S1** Yunkang oral liquid

**Table S1. table with each articles.**

| **articles** | **product information** | **Source** | **composition statement** | **Quality control reported** |
| --- | --- | --- | --- | --- |
| [1] Sadat SS, Zahra R, Hadi HN, Hossein R, Masoud M, Saeid A. Uterine Natural Killer Cell and Human Leukocyte Antigen-G1 and Human Leukocyte Antigen-G5 Expression in Vaginal Discharge of Threatened-Abortion Women: A Case-Control Study. J IMMUNOL RES. 2015: 692198. PMID: 4609863. | - | - | - | - |
| [2] Pengfei Z, Hang Z, Pei G, Wanting X, Jinzhu H, Qian Z,.Efficacy and safety of traditional Chinese herbal medicine in the treatment of threatened abortion: A protocol for systematic review and meta-analysis. Medicine.2021;100: e23288. PMID:7870177. | - | - | - | - |
| [3] Sapra KS, Joseph KS, Galea S, Bates LM, Buck Louis GM, Ananth CA, Signs and Symptoms of Early Pregnancy Loss: A Systematic Review. Reprod Sci. 2017;24: 502–513. PMID: 5933199. | - | - | - | - |
| [4] Ka CDM, Wang CK, Fei YSS, Yan LVC, Wun LRH, Yu NEH. A randomized double-blind controlled trial of the use of dydrogesterone in women with threatened miscarriage in the first trimester: study protocol for a randomized controlled trial. Trials. 2016;17: 408. PMID: 4989484. | - | - | - | - |
| [5] Giakoumelou S, Wheelhouse N, Cuschieri K, Entrican G, Howie SEM, Horne AW. The role of infection in miscarriage. HUM REPROD UPDATE. 2016;22: 116-133. PMID: 4664130. | - | - | - | - |
| [6] Ke RW. Endocrine Basis for Recurrent Pregnancy Loss. OBSTET GYN CLIN N AM. 2014 ;41: 103. | - | - | - | - |
| [7] Elisabeth P, Luc S, Gisèle M, Celine C, Caroline B, Florence B, et al. Hydroxychloroquine for prevention of recurrent miscarriage: study protocol for a multicentre randomised placebo-controlled trial BBQ study. BMJ Open. 2019;9: e025649. PMID: 6527997. | - | - | - | - |
| [8] Wai KC, C AJ, Min LS, Li CA, Soon TN, Chye TT. Serum progesterone distribution in normal pregnancies compared to pregnancies complicated by threatened miscarriage from 5 to 13 weeks gestation: a prospective cohort study. BMC PREGNANCY CHILDB. 2018;18: 360. PMID: 6126027. | - | - | - | - |
| [9] T. NS, G. NF, J. BG, M. G. Progesterone production in early pregnancy. Fertility and sterility. 1991; 55: 516-21. | - | - | - | - |
| [10] Yuan S, Gao F, Xin Z, Guo H, Shi S, Yang X, et al. Comparison of the efficacy and safety of phloroglucinol and magnesium sulfate in the treatment of threatened abortion: A meta-analysis of randomized controlled trials. Medicine (Baltimore). 2019;98: e16026. PMID: 6587576. | - | - | - | - |
| [11] Mei J, Ling H, Xiaohong G, Tiegang L, Jia K, Ting W. Traditional Chinese herb for low endometrial receptivity and its effect on pregnancy: Protocol for a systematic review and meta-analysis. Medicine (Baltimore). 2019;98: e17841. PMID: 6882586. | - | - | - | - |
| [12] National Pharmacopoeia Commission. Pharmacopoeia of the People's Republic of China Volume 1-859. China Medical Science and Technology Press; 2020. | Yunkang oral liquid | - | Shan yao 125g, Xu duan 75g, Huang qi 100g, Dang gui 75g, Gou ji(qumao) 100g, Tu si zi(bing) 75g, Sang ji sheng 50g, Du zhong(chao) 75g, Bu gu zhi 75g, Dang shen 75g, Fu ling 100g, Bai zhu(jiao) 75g, e’ jiao 25g, Di huang 100g, Shan zhu yu 75g, Gou qi zi 100g, Wu mei 50g, Bai shao 75g, Sha ren 50g, Yi zhi 50g, Zhu ma gen 75g, Huang qin 50g, Ai ye 8.3g. | - |
| [13] Li Zhen.Therapeutic observation of Yunkang oral liquid combined with chorionic gonadotropin in the treatment of threatened abortion.Modern Medicine and Clinical,2017,32:1310-1313. | Yunkang oral liquid | Echobe Group Zhejiang Qiqi Pharmaceutical Co., Ltd.  National medicine approval Z10970068 | The article doesn’t specify the composition of Yunkang oral liquid | Y-Pharmacopoeia of the People's Republic of China 2020 Edition I Formulations and single-flavor preparations |
| [14] Shi Qiuqiu, Yan Meiqiu, Yu Huanhuan, Chen Qingqing, Chen Suhong, Lv Guiyuan. Study on the effect of Yunkang oral liquid on fetal preservation and immune tolerance regulation of LPS-induced abortion in mice. China Journal of Traditional Chinese Medicine,2019,44:1227-1232 | Yunkang oral liquid | Echobe Group Zhejiang Qiqi Pharmaceutical Co., Ltd.  National medicine approval Z10970068 | The article doesn’t specify the composition of Yunkang oral liquid | Y-Pharmacopoeia of the People's Republic of China 2020 Edition I Formulations and single-flavor preparations |
| [15] Bo C, Qiu-Qiu S, Kai-Lun L,Yuyue X, Yingying F, Suhong C, et al. Effect and mechanism of Yunkang oral liquid in regulating endocrine system and VEGF signaling pathway and reducing abortion rate in recurrent abortion mice. Zhongguo Zhong yao za zhi 2018;43:1894-1900. | Yunkang oral liquid | Echobe Group Zhejiang Qiqi Pharmaceutical Co., Ltd.  National medicine approval Z10970068 | Shan yao 125g, Xu duan 75g, Huang qi 100g, Dang gui 75g, Gou ji(qumao) 100g, Tu si zi(bing) 75g, Sang ji sheng 50g, Du zhong(chao) 75g, Bu gu zhi 75g, Dang shen 75g, Fu ling 100g, Bai zhu(jiao) 75g, e’ jiao 25g, Di huang 100g, Shan zhu yu 75g, Gou qi zi 100g, Wu mei 50g, Bai shao 75g, Sha ren 50g, Yi zhi 50g, Zhu ma gen 75g, Huang qin 50g, Ai ye 8.3g. | Y-Pharmacopoeia of the People's Republic of China 2020 Edition I Formulations and single-flavor preparations |
| [16] Yang M, Luo JH, Li Y, Xu LM. Systems Pharmacology-Based Research on the Mechanism of Tusizi-Sangjisheng Herb Pair in the Treatment of Threatened Abortion. Biomed Res Int. 2020;2020: 4748264. PMID: 7391104. | - | - | - | - |
| [17] Sun L, Yuan Z, Jian L, Jiang QH, Zhang SW, Tan JC. The Modified Bushen Antai Recipe Upregulates Estrogen and Progesterone Receptors at the Maternal-Fetal Interface in Pregnant Rats with Mifepristone-Induced Pregnancy Loss. EVID-BASED COMPL ALT. 2019;2019: 8312020. PMID: 6354171. | - | - | - | - |
| [18] Liu MJ, Xiao GG, Rong PJ, Dong JZ, Zhang ZG, Zhao HY, et al. Semen astragali complanati- and rhizoma cibotii-enhanced bone formation in osteoporosis rats, BMC COMPLEM ALTERN M. 2013;13:B141 PMID: 3699375. | - | - | - | - |
| [19] Wu H., Ren C., Yang F., Qin Y., Zhang Y., Liu J. Extraction and identification of collagen-derived peptides with hematopoietic activity from Colla Corii Asini. J ETHNOPHARMACOL. 2016;182:129–136. | - | - | - | - |
| [20] Saraswat L, Bhattacharya S, Maheshwari A. Maternal and perinatal outcome in women with threatened miscarriage in the first trimester: a systematic review. BJOG. 2010;117:245–257. | - | - | - | - |
| [21] Huang XM, Wang J, Lin WJ,.Zhang N, Du JJ, Long Z, et al. Kanglaite injection plus platinum-based chemotherapy for stage III/IV non-small cell lung cancer: A meta-analysis of 27 RCTs. PHYTOMEDICINE. 2019;67: 153154. | - | - | - | - |

**Table S2. More details about the product information of Yunkang oral liquid**

| **Injection name** | **Source** | **Species**  **Raw materials** | **Chemical composition criteria** | **Therapeutic claims in TCM** | **Indications** | **Quality control reported? (Y/N)** | **Chemical analysis reported? (Y/N)** |
| --- | --- | --- | --- | --- | --- | --- | --- |
| Yunkang oral liquid | Echobe Group Zhejiang Qiqi Pharmaceutical Co., Ltd.  National medicine approval Z10970068 | Table S2 | Baicalin,  >0.8mg/1mL; Paeoniflorin,  >0.25mg/1mL; | Nourish the kidney and nourishing tocolysis | Threatened abortion, habitual abortion, infertility caused by abortion, etc. | Y-Pharmacopoeia of the People's Republic of China 2020 Edition I Formulations and single-flavor preparations | N |

**TABLE S3. Basic information of YKOL**

| Chinese botanical drugs | Latin name | Part of botanical drugs | Proportion |
| --- | --- | --- | --- |
| Shan yao | *Dioscorea opposita Thunb.* | rhizome | 125g |
| Xu duan | *Dipsacus asper Wall. Ex Henry* | root | 75g |
| Huang qi | *Astragalus membranaceus（Fisch.）Bge.* | root | 100g |
| Dang gui | *Angelica sinensis（Oliv.）Diels* | root | 75g |
| Gou ji(qumao) | *Cibotium barometz（L.）J.Sm.* | rhizome | 100g |
| Tu si zi(bing) | *Cuscuta australis R.Br.* | seed | 75g |
| Sang ji sheng | *Taxillus chinensis（DC.）Danser* | stalk | 50g |
| Du zhong(chao) | *Eucommia ulmoides Oliv.* | bark | 75g |
| Bu gu zhi | *Psoralea corylifoliaL.* | fruit | 75g |
| Dang shen | *Codonopsis pilosula (Franch.)Nannf.* | root | 75g |
| Fu ling | *Poria cocos（Schw.）Wolf* | sclerotium | 100g |
| Bai zhu(jiao) | *Atractylodes macrocephala Koidz.* | rhizome | 75g |
| e’jiao | *Equus asinus L.* | skin | 25g |
| Di huang | *[Rehmannia glutinosa (Gaertn.) DC.](https://mpns.science.kew.org/mpns-portal/plantDetail?plantId=527243&query=Prepared+Rehmannia+Root+&filter=&fuzzy=false&nameType=all&dbs=wcsCmp)* | rhizome | 100g |
| Shan zhu yu | *Cornus officinalis Sieb. et Zucc.* | fruit | 75g |
| Gou qi zi | *Lcycium barbarumL.* | fruit | 100g |
| Wu mei | *Prunus mume（Sieb.）Sieb.etZucc.* | fruit | 50g |
| Bai shao | *Paeonia lactiflora Pall.* | root | 75g |
| Sha ren | *Amomum villosum Lour.* | fruit | 50g |
| Yi zhi | *Alpinia oxyphylla Miq.* | fruit | 50g |
| Zhu ma gen | *Boehmeria nivea(L.)Gaud.* | root | 75g |
| Huang qin | *Scutellaria baicalensis Georgi* | root | 50g |
| Ai ye | *Artemisia argyi Levl.et Vant.* | foliage | 8.3g |

# The chemical characterisation of Yunkang oral liquid

The information comes from Pharmacopoeia of the People's Republic of China 2020 Edition I Formulations and single-flavor preparations.

**[Approval number]** National medicine approval Z10970068

**[The product name]** Yunkang oral liquid

**[Prescription]**

Shan yao 125g, Xu duan 75g, Huang qi 100g, Dang gui 75g, Gou ji(qumao) 100g,

Tu si zi(bing) 75g, Sang ji sheng 50g, Du zhong(chao) 75g, Bu gu zhi 75g, Dang shen 75g,

Fu ling 100g, Bai zhu(jiao) 75g, e’ jiao 25g, Di huang 100g, Shan zhu yu 75g, Gou qi zi 100g,

Wu mei 50g, Bai shao 75g, Sha ren 50g, Yi zhi 50g, Zhu ma gen 75g, Huang qin 50g, Ai ye 8.3g.

**[Preparation method]**

For the above twenty-three flavors, except donkey-hide gelatin, the other twenty-two flavors such as yam are soaked in warm water for 4 hours, filtered, and the filtrate is used for later use. hours, the third time for 0.5 hours, filtered, merged the above-mentioned filtrate, after adding donkey-hide gelatin to dissolve, concentrated into a clear paste containing 1 g of crude drug per 1ml; the clear paste was added with ethanol to make the alcohol content reach 70%, left standstill, filtered, and the filtrate was Recover ethanol, add 83 g of honey, 88 g of sucrose, 3.0 g of sodium benzoate and an appropriate amount of water, mix well, add sodium hydroxide test solution to adjust the pH value to 5~6, add water to 1000 ml, filter, encapsulate, sterilize, and get .

**[Properties]**

This product is a brown liquid; it is slightly odorous and sweet.

**[Identification]**

(1) Take 40ml of this product, shake and extract 3 times with butanone, 20ml each time, combine the extracts, evaporate to dryness, dissolve the residue in 5ml of methanol, add it to a neutral alumina column (100~200 mesh, 8g, inner diameter of 10~15mm), eluted with 150ml of 40% methanol, collected the eluate, evaporated to dryness, the residue was dissolved in 30ml of ammonia test solution, and extracted twice with water-saturated n-butanol by shaking. 10ml each time, the n-butanol solution was combined; washed once with 20ml of water, the aqueous solution was discarded, the n-butanol solution was evaporated to dryness, the residue was dissolved in 1ml of methanol, and used as the test solution. Another reference substance of astragaloside IV was taken, and methanol was added to make a solution containing 0.5 mg per 1 ml, which was used as the reference substance solution. According to the thin-layer chromatography (general rule 0502) test, draw 5μl of each of the above two solutions, and place them on the same silica gel G thin-layer plate respectively. The lower layer solution is a developing agent, which is developed, taken out, air-dried, sprayed with 10% sulfuric acid ethanol solution, heated at 105 ℃ until the spot color is clear. In the chromatogram of the test substance, the spots of the same color appear at the corresponding positions of the chromatogram of the reference substance; when viewed under an ultraviolet light (365 nm), the fluorescent spots of the same color appear.

(2) Take 20ml of this product, add 20ml of petroleum ether (30~60°C), shake and extract, separate the petroleum ether liquid, evaporate to dryness, add 1ml of ethyl acetate to the residue to dissolve it, and use it as the test solution. In addition, psoralen reference substance and isopsoralen reference substance were taken, and ethyl acetate was added to make a mixed solution containing 1 mg per 1 ml, which was used as a reference substance solution. According to the thin-layer chromatography (General Rule 0502) test, draw 5-10 μl of each of the above two solutions, and place them on the same silica gel G thin-layer plate respectively. Use n-hexane-ethyl acetate (4:1) as the developing solvent to develop, Take it out, dry it, spray it with 10% potassium hydroxide methanol solution, and inspect it under a UV lamp (365 nm). In the chromatogram of the test substance, there are fluorescent spots of the same color at the corresponding position of the chromatogram of the reference substance.

(3) Take this product and test it according to the method under [Determination of Content] Scutellaria baicalensis. The chromatographic peak of the test product should be consistent with the retention time of the chromatographic peak of the reference substance.

(4) Take this product and test it according to the method under [Determination of Content] Paeonia lactiflora. The chromatogram of the test product should present a chromatographic peak consistent with the retention time of the chromatographic peak of the reference substance.

**[Check]**

The relative density should not be less than 1.13 (General Rule 0601).

The pH value should be 4.5~6.0 (General Rule 0631).

Others should comply with the relevant provisions under the mixture (General Rule 0181).

**[Determination of content]**

【Determination of content】

**Scutellaria**

Determine according to high performance liquid chromatography (General Rule 0512).

**Chromatographic conditions and system suitability test**

Octadecylsilane-bonded silica gel was used as the filler; methanol-1% acetic acid solution (44:56) was used as the mobile phase; the detection wavelength was 278 nm. The number of theoretical plates should not be less than 2000 according to the baicalin peak.

**Preparation of reference solution**

Take an appropriate amount of baicalin reference substance, accurately weigh it, and add 50% methanol to make a solution containing 25μg per 1 ml.

**Preparation of test solution**

Precisely measure 1ml of this product, put it in a 50ml measuring bottle, add 50% methanol to dissolve and dilute to the mark, shake well, filter, and take the filtrate.

**Measurement method**

Precisely draw 10 μl of the reference solution and the test solution, respectively, and inject them into a liquid chromatograph for measurement.

Each 1ml of this product contains baicalin, calculated as baicalin (C_21_H_18_O_11_), not less than 0.80mg.

**white peony**

Determine according to high performance liquid chromatography (General Rule 0512).

**Chromatographic conditions and system suitability test**

Octadecylsilane-bonded silica gel was used as the filler; acetonitrile-0.1% phosphoric acid solution (14:86) was used as the mobile phase; the detection wavelength was 230 nm. The theoretical plate number should not be less than 2000 according to the paeoniflorin peak.

**Preparation of reference solution**

Take an appropriate amount of paeoniflorin reference substance, accurately weigh it, and add 50% methanol to make a solution containing 20 μg per 1 ml.

**Preparation of test solution**

Take the test solution under the item [Determination of Content] Scutellaria baicalensis.

**Measurement method**

Precisely draw 10 μl of the reference solution and 20 μl of the test solution, respectively, and inject them into a liquid chromatograph for determination.

Each 1ml of this product contains Paeoniflorin, calculated as Paeoniflorin (C23H28O11), not less than 0.25mg.

**[Functions and Indications]**

Threatened abortion, habitual abortion, infertility caused by abortion, etc.

**[Usage and Dosage]**

Oral. Take orally on an empty stomach in the morning, noon and evening, 20ml each time, 3 times a day.

**[Note]**

(1) During taking the medicine, avoid spicy and irritating food, avoid strenuous exercise and heavy physical labor.

(2) Any miscarriage, ectopic pregnancy, hydatidiform mole, etc. that are unavoidable are not applicable to this product.

**[Specifications]**

(1) 10ml per bottle (2) 20ml per bottle (3) 100ml per bottle

**[Storage]**

Shade, seal, store in a cool place.
