# Supplementary material for: Efficacy and safety of Yunkang oral liquid combined with conventional therapy for threatened miscarriage of first-trimester pregnancy a protocol for systematic review and meta-analysis
Source: PLoS One. 2022 Feb 8;17(2):e0263581. doi: 10.1371/journal.pone.0263581 (PMC8824317; doi:10.1371/journal.pone.0263581)
Supplement: Attachment — Submitted filename: PLOS ONE Efficacy and safety of Yunkang oral liquil.docx [file pone.0263581.s004.docx]

PLOS ONE Efficacy and safety of Yunkang oral liquil combined with conventional therapy for threatened miscarriage of first-trimester pregnancy A protocol for systematic review and meta-analysis

Methodological peer review Mia Schmidt-Hansen 2/9/21

Abstract:

There are a number of language issues, e.g.,

-“relevant randomized controlled trials (RCTs) were searched”. It is not the RCTs that are searched, but the databases.

-Past tense used in abstract in places. This needs to be changed to future tense.

Search date given is in the past (April 2021). A protocol should be published before any review work takes place to allow the peer review comments of the protocol to be taken into account in the actual work.

“The aim of this study was to assess the efficacy and safety of YKOL in the 12 treatment of threatened miscarriage during the first-trimester pregnancy (TMFP).” Please expand on the aim taking clear that this is a systematic review not a single study.

What are the inclusion criteria for the RCTs? Please include details of the population, interventions, comparators and outcomes.

What statistical analyses are you going to perform?

“After screening the literature based on the 19 inclusion and exclusion criteria, the Recommendation, Assessment, Development, and Evaluation (GRADE) 20 system will be used to evaluate the quality evidence of each result.” There are a number of steps missing between screening the literature and applying GRADE. Including the information requested above will go some what in addressing this gap, but the sentence need revising.

“Systematic review registration: INPLASY202140105.” What does this number refer to? Where is it registered? Why are you registering it here too? Due to the fact that you’ll have to make changes as a result of peer review, the protocol records will not be the same for the same review, so I don’t understand why you are going for this protocol publication?

Background:

What does “TMFP”, “YKOL” and “TKOL” mean?

“However, the systematic reviews examining the efficacy and safety of YKOL in the treatment of TMFP have 59 never been systematically evaluated.” This sentence contradicts itself. Has systematic reviews been done or not?

Methods:

Please describe the types of included studies more. Do they have to be parallel-design or can they be cross-over?

Please describe the participants more. What are the criteria for threatened miscarriage? You can reference the criteria you refer to, but they also need to be explicitly described. As an aside, I wonder why the women have to be 18 years? What if there is an otherwise eligible trial that has included women from 16 years? I recommend deleting the age criterion. You also need to describe the gestational age limits for inclusion.

The intervention needs to be much more described also, including how it is taken, how often, how much etc. What is conventional therapy? This also needs to be much more and better described. Are you effectively proposing to compare YKOL + standard care versus stand care alone?

Is the comparator effectively standard care or other active drugs? What about placebo?

Exclusion criteria: The test here needs extensive revision. It is a given that “1. The unrelated documents” will not be included, but “studies repeated publication” should not be deleted, but all publications of the same study should be used for data extraction, while of course treating the study as only one study”. It is also a given that “2. Semi-randomized controlled trials, retrospective studies experience summaries, case series, case reports, animal experiments and NARRATIVE reviews” will not be included. However, other systematic reviews should be checked for missed studies. Point 3 (“3. Review articles without incomplete data and have obvious errors.”) does not make sense, firstly because review articles do not contain original data and also secondly (and much less relevant as the sentence needs to be deleted) “without incomplete data” must be a typo?

Primary outcomes: Please describe in more detail. How will it be measured and what time points relative to the intervention are you interested in?

Secondary outcomes: I don’t know what they mean as outcome measures? The same comment as for the primary outcome applies here.

Search strategy: Same comments apply as on the abstract. Why is the end date 21/4/21? Why are you limiting the language to English and Chinese? Ideally, there should be no language or publication status limits, but if there are, a rationale for such limits need to included.

Why are you not searching trials databases and recent conference proceedings? You also need to check any relevant systematic reviews for missed studies, and explicitly state that you will do this.

My expertise is not in searching, but line 18 looks wrong of your search strategy (i.e., include animals not humans). Also, all the individual search strategies for each database need to be included in an appendix.

“Data screening Two independent researchers will screen the eligible studies according to the eligibility criteria and exclusion criteria, and disagreement in risk of bias will be resolved by three investigators through consultation. When there are disagreements, three researchers will resolve the disagreements. Details of the selection procedure for studies are shown in a PRISMA-P flow chart (Fig. 1)” There are a number of steps missing in this description to do with sifting the search on title and abstract and then full text. What is the relevance to risk of bias here? Please see a published Cochrane intervention review protocol and the Cochrane Intervention review Handbook for further guidance.

Data extraction and management: The choice of words here make it difficult to understand. What is “filtered” articles? What is “ultimate eligible studies”? In terms of the data that will be extracted, then this also needs to be revised and clarified, but what is the relevance of the first author’s country? Surely the country the study was conducted in is the relevant country to extract. What does diagnostic criteria, treatment period, time of pregnancy, basic therapies; treatment and control measurements: outcomes: baseline and follow-up data mean in this regard? Be explicit and clear.

Quality assessment: You need to state which checklist you will use to assess risk of bias. Review Manager is not a checklist. In your description of the checklist you also need to use the same words that it uses rather than for example “whether distribution is hidden”. You also need to explicitly detail which items will be assessed at the study level and which will be assessed at the outcome level.

Statistical analysis: What will determine whether you use odds ratios or relative risks? What will determine whether you analyse mean differences or standardised mean differences? What will you do if the Q value test and I2 indicate statistical heterogeneity? What criteria will you use to determine whether there is heterogeneity? Why are you planning to use a random effects model and not fixed effects?

Dealing with missing data: What is “elusive” data?

Subgroup analysis: What is “substantial heterogeneity”? What does “the grouping factor for Subgroup analysis will be executed according to therapy time and number of abortions” mean?

Sensitivity analysis: How will you “change the statistical model”?

Quality of evidence: How will you apply GRADE to your data? You need to detail what will make you downgrade 1 or 2 levels for each of the GRADE domains.

A protocol should not include a discussion. Please delete.
